# Supplementary material for: Leptin receptor+ cells promote bone marrow innervation and regeneration by synthesizing nerve growth factor
Source: Nat Cell Biol. 2023 Nov 27;25(12):1746–57. doi: 10.1038/s41556-023-01284-9 (PMC10709146; doi:10.1038/s41556-023-01284-9)
Supplement: Supplementary file 1 — Reporting Summary [file 41556_2023_1284_MOESM1_ESM.pdf]

nature portfolio

Corresponding author(s): Bo, Shen, Sean Morrison

Last updated by author(s): Sep 19, 2023

Reporting Summary

Nature Portfolio wishes to improve the reproducibility of the work that we publish. This form provides structure for consistency and transparency in reporting. For further information on Nature Portfolio policies, see our Editorial Policies and the Editorial Policy Checklist.

Statistics

For all statistical analyses, confirm that the following items are present in the figure legend, table legend, main text, or Methods section.

n/a	Confirmed	
☐	☒	The exact sample size (*n*) for each experimental group/condition, given as a discrete number and unit of measurement
☐	☒	A statement on whether measurements were taken from distinct samples or whether the same sample was measured repeatedly
☐	☒	The statistical test(s) used AND whether they are one- or two-sided<br>*Only common tests should be described solely by name; describe more complex techniques in the Methods section.*
☒	☐	A description of all covariates tested
☐	☒	A description of any assumptions or corrections, such as tests of normality and adjustment for multiple comparisons
☐	☒	A full description of the statistical parameters including central tendency (e.g. means) or other basic estimates (e.g. regression coefficient) AND variation (e.g. standard deviation) or associated estimates of uncertainty (e.g. confidence intervals)
☐	☒	For null hypothesis testing, the test statistic (e.g. *F*, *t*, *r*) with confidence intervals, effect sizes, degrees of freedom and *P* value noted<br>*Give P values as exact values whenever suitable.*
☒	☐	For Bayesian analysis, information on the choice of priors and Markov chain Monte Carlo settings
☒	☐	For hierarchical and complex designs, identification of the appropriate level for tests and full reporting of outcomes
☒	☐	Estimates of effect sizes (e.g. Cohen's *d*, Pearson's *r*), indicating how they were calculated

Our web collection on statistics for biologists contains articles on many of the points above.

Software and code

Policy information about availability of computer code

Data collection	Flow cytometry data were collected using BD FACSDiva 8.0. Confocal images were acquired using Zeiss Zen-2, or Leica Application Suite X 4.6.1.27508. Bulk and single-cell RNA-seq data were obtained from previous published studies.
Data analysis	GraphPad Prism V10.0 and R 4.0.2 with the stats, fBasics 4021.93, car 3.0-10, and Seurat 4.3.0 packages for bioinformatic and statistical analyses. Flow cytometry data analysis using BD FACSDiva 8.0, and FlowJo V10 (Treestar). Confocal images were processed and analysed using Zeiss Zen-2, or rendered in 3D and analysed using either Bitplane Imaris v7.7.1 or Aivia 10.5.1 software,

For manuscripts utilizing custom algorithms or software that are central to the research but not yet described in published literature, software must be made available to editors and reviewers. We strongly encourage code deposition in a community repository (e.g. GitHub). See the Nature Portfolio guidelines for submitting code & software for further information.

Data

Policy information about availability of data

All manuscripts must include a data availability statement. This statement should provide the following information, where applicable:

- Accession codes, unique identifiers, or web links for publicly available datasets
- A description of any restrictions on data availability
- For clinical datasets or third party data, please ensure that the statement adheres to our policy

Microscopy and flow cytometry data reported in this paper will be shared by the lead contacts upon reasonable request. Published microarray data that were re-

analyzed in this study are available in the National Center for Biotechnology Information (NCBI) GEO database under accession code GSE33158. Published bulk and single cell RNA sequencing data that were re-analyzed in this study are available in the NCBI BioProject database under accession codes PRJNA914703 and PRJNA835050. This paper does not report original code. Source data are provided with this study. All other data supporting the findings of this study are available from the corresponding authors on reasonable request.

Human research participants

Policy information about studies involving human research participants and Sex and Gender in Research.

Reporting on sex and gender	N/A
Population characteristics	N/A
Recruitment	N/A
Ethics oversight	N/A

Note that full information on the approval of the study protocol must also be provided in the manuscript.

Field-specific reporting

Please select the one below that is the best fit for your research. If you are not sure, read the appropriate sections before making your selection.

☒ Life sciences ☐ Behavioural & social sciences ☐ Ecological, evolutionary & environmental sciences

For a reference copy of the document with all sections, see nature.com/documents/nr-reporting-summary-flat.pdf

Life sciences study design

All studies must disclose on these points even when the disclosure is negative.

Sample size	Samples sizes were not pre-determined based on statistical power calculations but our sample sizes are similar to those reported in previous publications (Nature 591:438, Nat Cell Biol 19:891, Nat Med 19:695).
Data exclusions	No data were excluded.
Replication	To ensure reproducibility, multiple independent biological replicates were performed with numbers of independent experiments specified in each figure legend. We did not exclude data from unsuccessful replication attempts.
Randomization	Mice were allocated to experiments randomly and samples processed in an arbitrary order, but formal randomization techniques were not used.
Blinding	No formal blinding was applied when performing the experiments or analyzing the data. Blinding was not performed during data collection or analysis.

Reporting for specific materials, systems and methods

We require information from authors about some types of materials, experimental systems and methods used in many studies. Here, indicate whether each material, system or method listed is relevant to your study. If you are not sure if a list item applies to your research, read the appropriate section before selecting a response.

Materials & experimental systems

n/a	Involved in the study	
☐	☒	Antibodies
☒	☐	Eukaryotic cell lines
☒	☐	Palaeontology and archaeology
☐	☒	Animals and other organisms
☒	☐	Clinical data
☒	☐	Dual use research of concern

Methods

n/a	Involved in the study	
☒	☐	ChIP-seq
☐	☒	Flow cytometry
☒	☐	MRI-based neuroimaging

Antibodies

Antibodies used

The following antibodies have been used in this study:
Anti-mouse CD2, FITC, clone RM2-5, Cat. #35-0021, LOT: C0021121118354, Tonbo, 1:200, Flow cytometry and Immunofluorescence analysis

Anti-mouse CD3, FITC, clone 17A2, Cat. #100204, LOT: B304392, BioLegend, 1:200, Flow cytometry and Immunofluorescence analysis

Anti-mouse CD5, FITC, clone 53-7.3, Cat. #100606, LOT: B210716, BioLegend, 1:200, Flow cytometry and Immunofluorescence analysis

Anti-mouse CD8a, FITC, clone 53-6.7, Cat. #35-0081, LOT: C0081100219354, Tonbo, 1:200, Flow cytometry and Immunofluorescence analysis

Anti-mouse Ter119, FITC, clone TER-119, Cat. #116206, LOT: B272256, BioLegend, 1:200, Flow cytometry and Immunofluorescence analysis

Anti-mouse B220, FITC, clone RA3-6B2, Cat. #11-0452-86, LOT: 436128, eBiosciences, 1:200, Flow cytometry and Immunofluorescence analysis

Anti-mouse Gr-1, FITC, clone RB6-8C5, Cat. #35-5931, LOT: C5931080318354, Tonbo, 1:200, Flow cytometry and Immunofluorescence analysis

Anti-mouse c-Kit, APC-eFluor780, clone 2B8, Cat. #47-1171-82, LOT: 2018834, eBiosciences, 1:200, Flow cytometry

Anti-mouse Sca-1, PerCP-Cyanine5.5, clone D7, Cat. # 45-5981-82, LOT: 2162718, EBiosciences, 1:200, Flow cytometry

Anti-mouse CD150, PE, clone TC15-12F12.1, Cat. #115904, LOT: B270365, BioLegend, 1:200, Flow cytometry

Anti-mouse CD48, Alexa Fluor-700, clone HM48-1, Cat. #103426, LOT: B279067, BioLegend, 1:200, Flow cytometry

Anti-mouse CD127, PE-Cyanine7, clone A7R34, Cat. #60-1271, LOT: C1271121219603, Tonbo, 1:200, Flow cytometry

Anti-mouse CD135, APC, clone A2F10, Cat. #135310, LOT: B269023, BioLegend, 1:200, Flow cytometry

Anti-mouse CD34, Biotin, clone RAM34, Cat. #13-0341-85, LOT: 2075112, eBiosciences, 1:200, Flow cytometry

Anti-mouse CD16/32, BV510, clone 93, Cat. #101333, LOT: B303788, BioLegend, 1:200, Flow cytometry

Anti-mouse CD41, Alexa Fluor-700, clone /MWReg30, Cat. #133926, LOT: B270215, BioLegend, 1:200, Flow cytometry

Anti-mouse CD105, APC, clone MJ7/18, Cat. #120414, LOT: B266785, BioLegend, 1:200, Flow cytometry

Anti-mouse Gr-1, PE-Cyanine7, clone RB6-8C5, Cat. #108416, LOT: B284962, BioLegend, 1:200, Flow cytometry

Anti-mouse CD11b, APC-eFluor780, clone M1/70, Cat. #47-0112-82, LOT: 2011193, EBiosciences, 1:200, Flow cytometry

Anti-mouse B220, PerCP-Cyanine5.5, clone RA3-6B2, Cat. #65-0452, LOT: C0452060619653, Tonbo, 1:200, Flow cytometry

Anti-mouse IgM, APC, clone 11/41, Cat. #17-5790-82, LOT: 2167008, eBiosciences, 1:200, Flow cytometry

Anti-mouse CD43, PE, clone S7, Cat. #553271, LOT: 9336727, Fisher Scientific, 1:200, Flow cytometry

Anti-mouse CD3, redFluor-710, clone 17A2, Cat. #80-0032, LOT: C0032010319803, Tonbo, 1:200, Flow cytometry

Anti-mouse Ter119, BV510, clone TER-119, Cat. #116237, LOT: B270148, BioLegend, 1:200, Flow cytometry

Anti-mouse CD71, FITC, clone R17217, Cat. #11-0711-82, LOT: 2159109, eBiosciences, 1:200, Flow cytometry

Anti-mouse CD31, FITC, clone 390, Cat. #11-0311-82, LOT: 2086274, eBiosciences, 1:200, Flow cytometry

Anti-mouse CD144, eFluor-660, clone eBioBV13, Cat. #50-1441-82, LOT: 2007696, eBiosciences, 10 ug/mouse, Flow cytometry

Anti-mouse Peripherin, primary antibody, Cat. #ab4666, LOT: GR3383934-9, Abcam,1:250, Immunofluorescence analysis

Anti-mouse Leptin receptor, Biotin, Cat. #BAF497, LOT: BFV0719071, R&D Systems, 1:200, Flow; 1:200, Immunofluorescence analysis

Anti-mCherry, Cat. #632496, LOT: 1904182, Takara, 1:200, Immunofluorescence analysis

Cy3-conjugated AffiniPure Fab fragment donkey anti-rabbit IgG, Cat. #711-167-003, LOT: 145173, Jackson ImmunoResearch, 1:250, Immunofluorescence analysis

Alexa Fluor-488-conjugated AffiniPure Fab fragment donkey anti-chicken IgG, Cat. #703-546-155, LOT: 144594, Jackson ImmunoResearch, 1:250, Immunofluorescence analysis

PE-Cyanine7 streptavidin, Cat. #557598, LOT: 9011715, BD Biosciences, 1:500, Flow cytometry

BV421 streptavidin, Cat. #405226, LOT: B286541, BioLegend, 1:500, Flow cytometry

Anti-Green Fluorescent Protein Antibody, Cat. #GFP-1020, LOT: GFP3717982, Aves Labs, 1:250, Immunofluorescence analysis

Donkey anti-Goat IgG (H+L) Cross-Adsorbed Secondary Antibody, Alexa Fluor-555, Cat. #A-21432, LOT:2400919, Life Technologies, 1:500, Immunofluorescence analysis

Anti-tdTomato, Cat. #LS-C340696, LOT: 200314, LSBio, 1:500, Immunofluorescence analysis

Anti-Endomucin, Cat. #AF4666, LOT: CAAS0222041, R&D Systems, 1:250, Immunofluorescence analysis

Anti-Laminin, Cat. #ab7463, LOT: GR3408983-4, 1:250, Immunofluorescence analysis

Anti-S100 beta, Cat. #ab52642, LOT: GR3215095-26, 1:250, Immunofluorescence analysis

Anti-Perilipin, Cat.#P1873, LOT: 0000149699, 1:1000, Immunofluorescence analysis

Anti-Actin, α-Smooth Muscle-FITC antibody, Cat: F3777, LOT: 0000122364, 1:250, Immunofluorescence analysis

Alexa Fluor-488 AffiniPure F(ab')2 Fragment Donkey Anti-Rabbit IgG (H+L). Cat.#711-546-152. LOT: 161669, 1:250, Immunofluorescence analysis

Validation

All antibodies are commercially available and have been validated in previously published studies (e.g. Nature 495:231, Nature 591:438). We have independently validated antibodies that were central to our conclusions. For instance, the anti-LepR antibody was validated using mouse bone marrow cells deficient for Lepr (Cell Stem Cell 15:154).

Anti-mouse CD2. This monoclonal antibody recognizes mouse CD2. https://tonbobio.com/products/fitc-anti-mouse-cd2-rm2-5

Anti-mouse CD3. This monoclonal antibody recognizes mouse CD3. https://www.biolegend.com/en-us/products/fitc-anti-mouse-cd3-antibody-45

Anti-mouse CD5. This monoclonal antibody recognizes mouse CD5. https://www.biolegend.com/en-us/products/fitc-anti-mouse-cd5-antibody-159

Anti-mouse CD8a. This monoclonal antibody recognizes mouse CD8. https://tonbobio.com/products/fitc-anti-mouse-cd8a-53-6-7

Anti-mouse Ter119. This monoclonal antibody recognizes mouse Ter119. https://www.biolegend.com/en-us/products/fitc-anti-mouse-ter-119-erythroid-cells-antibody-1865

Anti-mouse B220. This monoclonal antibody recognizes mouse B220. https://www.thermofisher.com/antibody/product/CD45R-B220-Monoclonal-Antibody-RA3-6B2-FITC-eBioscience/11-0452-86

Anti-mouse Gr-1. This monoclonal antibody recognizes mouse Gr-1. https://tonbobio.com/products/fitc-anti-mouse-ly-6g-gr-1-rb6-8c5

Anti-mouse c-kit. This monoclonal antibody recognizes mouse c-kit. https://www.thermofisher.com/antibody/product/CD117-c-Kit-Antibody-clone-2B8-Monoclonal/47-1171-82

Anti-mouse Sca-1. This monoclonal antibody recognizes mouse Sca-1. https://www.thermofisher.com/antibody/product/Ly-6A-E-Sca-1-Antibody-clone-D7-Monoclonal/45-5981-82

Anti-mouse CD150. This monoclonal antibody recognizes mouse CD150. https://www.biolegend.com/en-us/products/pe-anti-mouse-cd150-slam-antibody-1369

Anti-mouse CD48. This monoclonal antibody recognizes mouse CD48. https://www.biolegend.com/en-us/products/alexa-fluor-700-anti-mouse-cd48-antibody-6670

Anti-mouse CD127. This monoclonal antibody recognizes mouse CD127. https://tonbobio.com/products/pe-cyanine7-anti-mouse-cd127-il-7ra-a7r34

Anti-mouse CD135. This monoclonal antibody recognizes mouse CD135. https://www.biolegend.com/en-us/products/apc-anti-mouse-cd135-antibody-6284

Anti-mouse CD34. This monoclonal antibody recognizes mouse CD34. https://www.thermofisher.com/antibody/product/CD34-Antibody-clone-RAM34-Monoclonal/13-0341-85

Anti-mouse CD16/32. This monoclonal antibody recognizes mouse CD16/32. https://www.biolegend.com/en-us/products/brilliant-violet-510-anti-mouse-cd16-32-antibody-9917

Anti-mouse CD41. This monoclonal antibody recognizes mouse CD41. https://www.biolegend.com/en-us/products/alexa-fluor-700-anti-mouse-cd41-antibody-13058

Anti-mouse CD105. This monoclonal antibody recognizes mouse CD105. https://www.biolegend.com/en-us/products/apc-anti-mouse-cd105-antibody-6519

Anti-mouse Gr-1. This monoclonal antibody recognizes mouse Gr-1. https://www.biolegend.com/en-us/products/pe-cy7-anti-mouse-ly-6g-ly-6c-gr-1-antibody-1931

Anti-mouse CD11b. This monoclonal antibody recognizes mouse CD11b. https://www.thermofisher.com/antibody/product/CD11b-Antibody-clone-M1-70-Monoclonal/47-0112-82

Anti-mouse B220. This monoclonal antibody recognizes mouse B220. https://tonbobio.com/products/percp-cyanine5-5-anti-human-mouse-cd45r-b220-ra3-6b2

Anti-mouse IgM. This monoclonal antibody recognizes mouse IgM. https://www.thermofisher.com/antibody/product/IgM-Antibody-clone-II-41-Monoclonal/17-5790-82

Anti-mouse CD43. This monoclonal antibody recognizes mouse CD43. https://www.fishersci.com/shop/products/cd43-rat-anti-mouse-pe-clone-s7-bd/bdb553271?matchedCatNo=BDB553271&searchHijack=true&searchTerm=BDB553271&searchType=RAPID

Anti-mouse CD3. This monoclonal antibody recognizes mouse CD3. https://tonbobio.com/products/redfluor-710-anti-mouse-cd3-17a2

Anti-mouse CD71. This monoclonal antibody recognizes mouse CD71. https://www.thermofisher.com/antibody/product/CD71-Transferrin-Receptor-Antibody-clone-R17217-RI7-217-1-4-Monoclonal/11-0711-82

Anti-mouse CD31. This monoclonal antibody recognizes mouse CD31. https://www.thermofisher.com/antibody/product/CD31-PECAM-1-Antibody-clone-390-Monoclonal/11-0311-82

Anti-mouse CD144. This monoclonal antibody recognizes mouse CD144. https://www.thermofisher.com/antibody/product/CD144-VE-cadherin-Antibody-clone-eBioBV13-BV13-Monoclonal/50-1441-82

Anti-mouse Peripherin. This polyclonal antibody recognizes mouse Peripherin. https://www.abcam.com/peripherin-antibody-ab4666.html

Anti-mouse Leptin Receptor. This monoclonal antibody recognizes mouse Leptin Receptor. https://www.rndsystems.com/products/mouse-leptin-r-biotinylated-antibody_baf497

Anti-mCherry. This polyclonal antibody recognizes tdTomato. https://www.takarabio.com/products/antibodies-and-elisa/fluorescent-protein-antibodies/red-fluorescent-protein-antibodies?catalog=632496

Cy3-conjugated AffiniPure Fab fragment donkey anti-rabbit IgG. This Fab fragment antibody was generated by papain digestion of whole IgG antibodies to remove the entire Fc portion, including the hinge region. This antibody is monovalent, containing only a single antigen binding site. Based on antigen-binding assay and/or ELISA, the antibody reacts with rabbit IgG. It also reacts with the light chains of other rabbit immunoglobulins. No binding was detected against non-immunoglobulin serum proteins. https://www.jacksonimmuno.com/catalog/products/711-167-003

Alexa Fluor-488-conjugated AffiniPure Fab fragment donkey anti-chicken IgG. This F(ab')2 fragment antibody was generated by pepsin digestion of whole IgG antibodies to remove most of the Fc region while leaving some of the hinge region. F(ab')2 fragments have two antigen-binding Fab portions linked together by disulfide bonds and therefore they are divalent. It is used for specific applications, such as to avoid binding of secondary antibodies to live cells with Fc receptors or to Protein A or Protein G. Based on immunoelectrophoresis and/or ELISA, the antibody reacts with whole molecule chicken IgY. It also reacts with the light chains of other chicken immunoglobulins. No binding was detected against non-immunoglobulin serum proteins. https://www.jacksonimmuno.com/catalog/products/703-546-155

PE-Cyanine7 streptavidin. This is a second-step reagent for the indirect immunofluorescent staining of cells in combination with biotinylated primary antibodies for flow cytometric analysis. https://www.bdbiosciences.com/us/reagents/research/antibodies-buffers/second-step-reagents/avidinstreptavidin/pe-cy7-streptavidin/p/557598

BV421 streptavidin. This is a second-step reagent for the indirect immunofluorescent staining of cells in combination with biotinylated primary antibodies for flow cytometric analysis. https://www.biolegend.com/en-us/products/brilliant-violet-421-streptavidin-7297
Anti-Green Fluorescent Protein. This polyclonal antibody recognizes mouse Peripherin. https://www.aveslabs.com/products/anti-green-fluorescent-protein-antibody-gfp

Donkey anti-Goat IgG (H+L) Cross-Adsorbed Secondary Antibody, Alexa Fluor-555. To minimize cross-reactivity, these donkey anti-goat IgG (H+L) whole secondary antibodies have been affinity purified and cross-adsorbed against rabbit, rat, mouse, and human IgG. Cross-adsorption or pre-adsorption is a purification step to increase specificity of the antibody resulting in higher sensitivity and less background staining. The secondary antibody solution is passed through a column matrix containing immobilized serum proteins from potentially cross-reactive species. Only the nonspecific-binding secondary antibodies are captured in the column, and the highly specific secondaries flow through. The benefits of this extra step are apparent in multiplexing/multicolor-staining experiments (e.g., flow cytometry) where there is potential cross-reactivity with other primary antibodies or in tissue/cell fluorescent staining experiments where there are may be the presence of endogenous immunoglobulins.
https://www.thermofisher.com/antibody/product/Donkey-anti-Goat-IgG-H-L-Cross-Adsorbed-Secondary-Antibody-Polyclonal/A-21432

Anti-tdTomato. This polyclonal antibody recognizes mouse tdtomato.
https://www.lsbio.com/antibodies/tdtomato-antibody-if-immunofluorescence-ihc-wb-western-ls-c340696/351334

Anti-Endomucin. This polyclonal antibody recognizes mouse Endomucin.
https://www.rndsystems.com/cn/products/mouse-endomucin-antibody_af4666#product-details

Anti-Laminin. This antibody is pan-specific and reacts well with all laminin isoforms tested: laminin-1 (alpha-1, beta-1, and gamma-1) and laminin-2 (alpha-2, beta-1, and gamma-1).
https://www.abcam.cn/products/primary-antibodies/laminin-12-antibody-ab7463.html

Anti-S100 beta. This polyclonal antibody recognizes mouse S100 beta.
https://www.abcam.cn/products/primary-antibodies/s100-beta-antibody-ep1576y-astrocyte-marker-ab52642.html

Anti-Perilipin. This polyclonal antibody recognizes mouse Perilipin.
https://www.sigmaaldrich.com/HK/en/product/sigma/p1873

Anti-Actin, α-Smooth Muscle-FITC. Monoclonal Anti-Actin, α-Smooth Muscle specifically recognizes the α-smooth muscle isoform of actin (42 kDa) by ELISA and immunoblotting.[2] It does not react with the other major actin isoforms present in fibroblasts or epithelial cells (β and γ-cytoplasmic), striated muscle (α-sarcomeric), myocardium (α-myocardial), or γ-smooth muscle isoform.
https://www.sigmaaldrich.com/HK/en/product/sigma/f3777

Alexa Fluor-488 AffiniPure F(ab')2 Fragment Donkey Anti-Rabbit IgG (H+L). F(ab')2 fragment antibodies are generated by pepsin digestion of whole IgG antibodies to remove most of the Fc region while leaving some of the hinge region. F(ab')2 fragments have two antigen-binding Fab portions linked together by disulfide bonds and therefore they are divalent. The average molecular weight is about 110 kDa. They are used for specific applications, such as to avoid binding of secondary antibodies to live cells with Fc receptors or to Protein A or Protein G.
https://www.jacksonimmuno.com/catalog/products/711-546-152

Anti-mouse Ter119. This monoclonal antibody recognizes mouse Ter119. https://www.biolegend.com/en-us/products/brilliant-violet-510-anti-mouse-ter-119-erythroid-cells-antibody-8243

Anti-Green Fluorescent Protein Antibody. This polyclonal antibody recognizes mouse Green Fluorescent Protein. https://www.aveslabs.com/products/anti-green-fluorescent-protein-antibody-gfp

Animals and other research organisms

Policy information about studies involving animals; ARRIVE guidelines recommended for reporting animal research, and Sex and Gender in Research

Laboratory animals	Lepr-cre (JAX Strain #:008320), Adiponectin-CreER (JAX Strain #:024671), NG2-DsRed (JAX Strain #:008241), NG2-CreER (JAX Strain #:008538), Col1a1-CreER (JAX Strain #:016241), GFAP-Cre (JAX Strain #:024098), Rosa26-CAG-loxp-stop-loxp-tdTomato (Ai14; JAX Strain #:007914), Rosa26-CAG-loxp-stop-loxp-EGFP (Ai47; previously published in Cell 174:465), Col1a1*2.3-EGFP (JAX Strain #:013134), ScfGFP (JAX Strain #:017860), Ngf null (previously published in Cell 76:1001), Adrb1 null (derived from JAX Strain #:003810), Adrb2 null (derived from JAX Strain #:003810), and Adrb3 null mice (JAX Strain #:006402) were previously characterized and used in this study. Ngf-mScarlet, Ngf floxed, Adrb2 floxed, and Adrb3 floxed mice were generated in this study. All mice were maintained on a C57BL/6J background, and two-month to 8-month-old mice were used.
Wild animals	No wild animals were used.
Reporting on sex	Random sex assignment was used in each experiment and findings apply to both sexes.
Field-collected samples	No field-collected samples were used.
Ethics oversight	All mouse experiments complied with all relevant ethical regulations and were performed according to protocols approved by the Institutional Animal Care and Use Committee at UT Southwestern Medical Center (UTSW; protocol 2017-101896) and the National Institute of Biological Sciences, Beijing (NIBS; protocol NIBS2022M0024). Mice were maintained under pathogen-free conditions of a 12 h light/dark cycle at controlled temperature (20-25 °C) and humidity (50-70%), and were provided with food and water ad libitum.

Note that full information on the approval of the study protocol must also be provided in the manuscript.

Flow Cytometry

Plots

Confirm that:

☒ The axis labels state the marker and fluorochrome used (e.g. CD4-FITC).

☒ The axis scales are clearly visible. Include numbers along axes only for bottom left plot of group (a 'group' is an analysis of identical markers).

☒ All plots are contour plots with outliers or pseudocolor plots.

☒ A numerical value for number of cells or percentage (with statistics) is provided.

Methodology

Sample preparation	Bone marrow hematopoietic cells were isolated by flushing the long bones with Ca2+- and Mg2+- free HBSS (HBSS-free) with 2% heat-inactivated bovine serum. Spleen cells were obtained by crushing the spleen between two glass slides. The cells were dissociated into a single cell suspension by gently passing them through a 25-gauge needle and then filtering through 70-um nylon mesh.<br><br>For flow cytometric analysis of stromal cells, bone marrow was flushed using HBSS-free with 2% bovine serum. Then whole bone marrow was digested with type I collagenase (3mg/ml), dispase (4mg/ml) and DNase I (1U/ml) at 37°C for 30 min. Samples were then stained with antibodies and analyzed by flow cytometry.
Instrument	BD FACS Aria Fusion (for cell sorting or analysis), BD Canto (for analysis).
Software	BD FACSDiva 8.0, FlowJo V10
Cell population abundance	The abundance of the relevant cell populations within post-sort fractions was 90-100% in experiments.
Gating strategy	Flow cytometry gating strategy for the isolation of hematopoietic stem and progenitor cell populations, LepR+ cells and endothelial cells were shown in Extended Data Fig. 2. To eliminate dead cells from sorts and analyses, cells were stained with 4’,6-diamidino-2-phenylindole (DAPI).

☒ Tick this box to confirm that a figure exemplifying the gating strategy is provided in the Supplementary Information.
