# Supplementary figures and images for: Leptin receptor+ cells promote bone marrow innervation and regeneration by synthesizing nerve growth factor
Source: Nat Cell Biol. 2023 Nov 27;25(12):1746–57. doi: 10.1038/s41556-023-01284-9 (PMC10709146; doi:10.1038/s41556-023-01284-9)

Unprocessed images in Figure 5

O

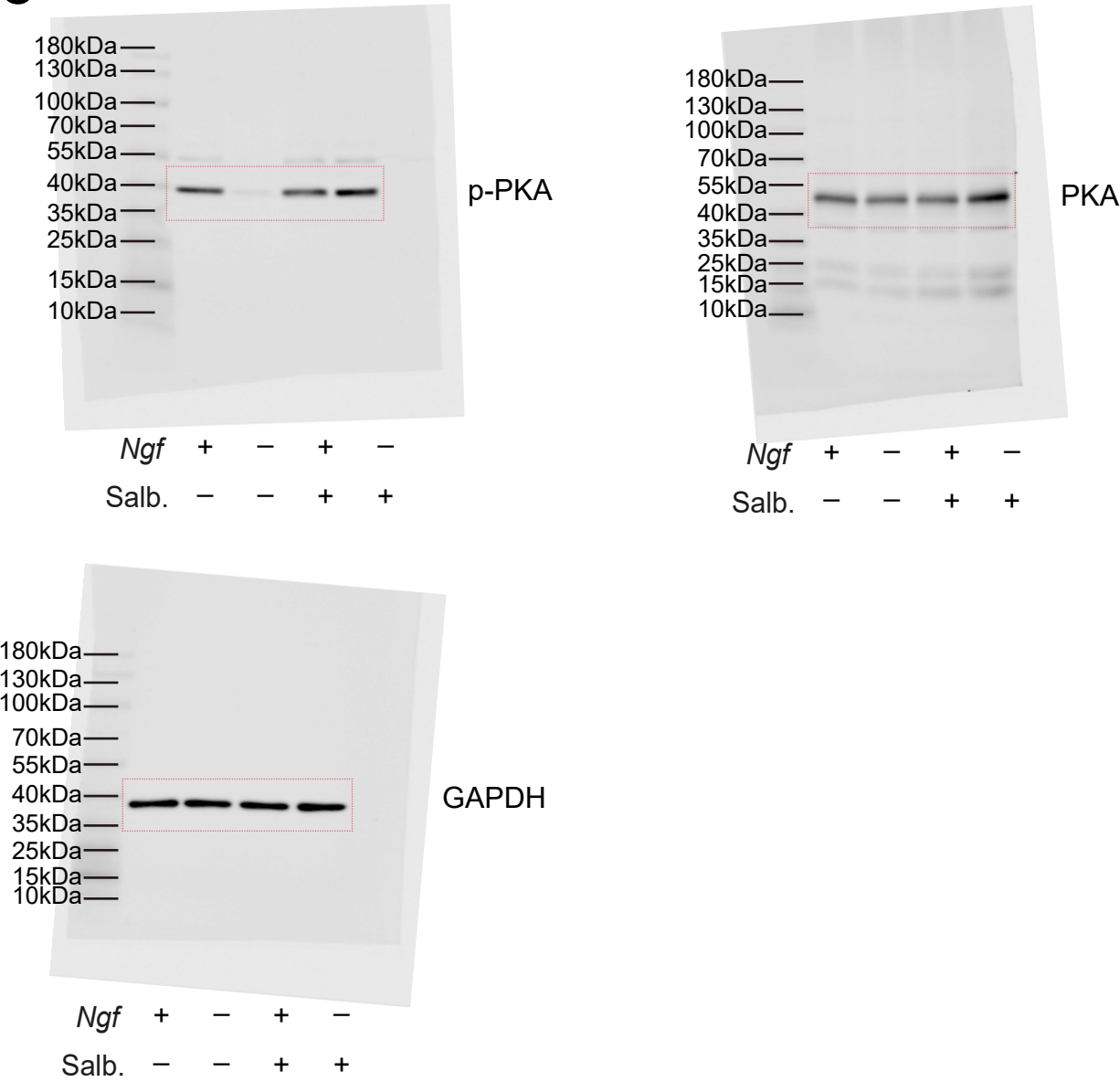

Supplement: Supplementary file 8 — Unprocessed images. [file 41556_2023_1284_MOESM8_ESM.pdf]

Unprocessed images in Figure 6

r

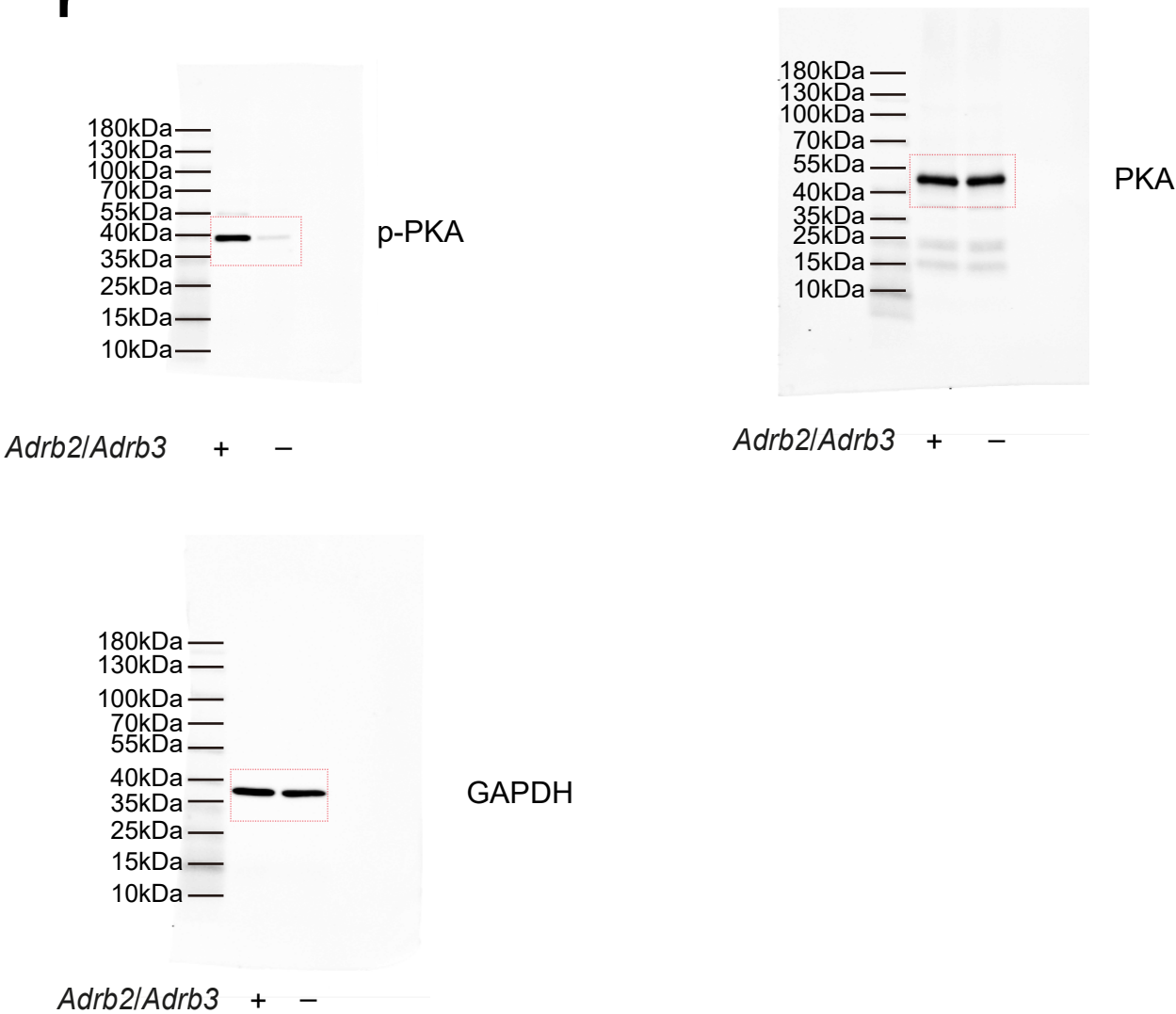

Supplement: Supplementary file 10 — Unprocessed images. [file 41556_2023_1284_MOESM10_ESM.pdf]

Unprocessed images in Extended Data Figure 1

**b**

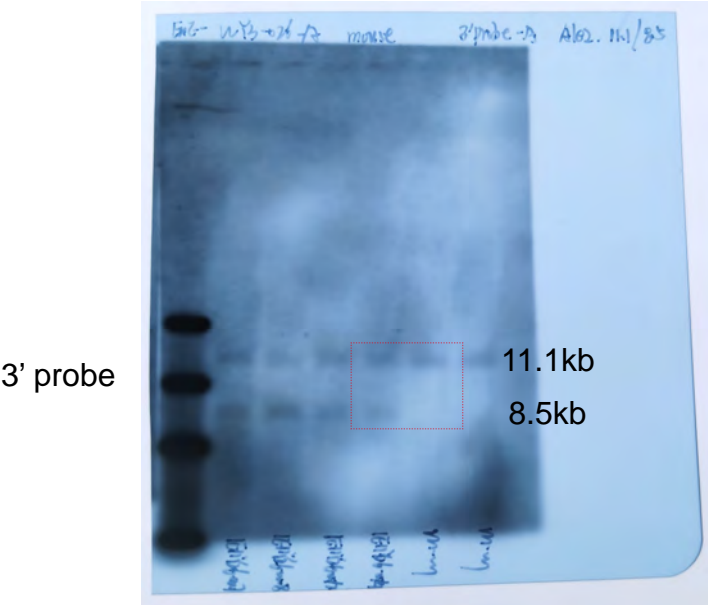

WPRE probe

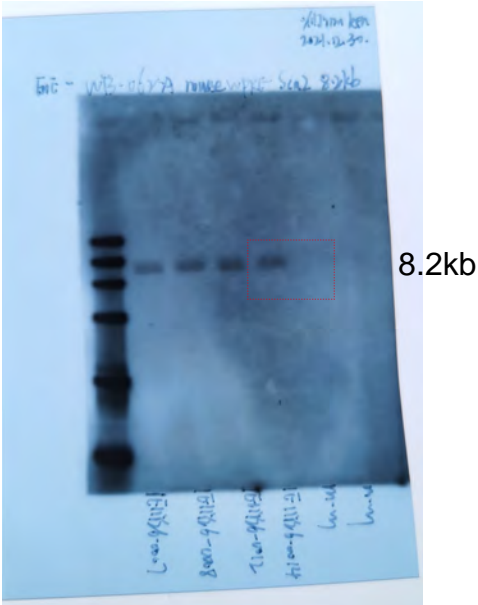

**c**

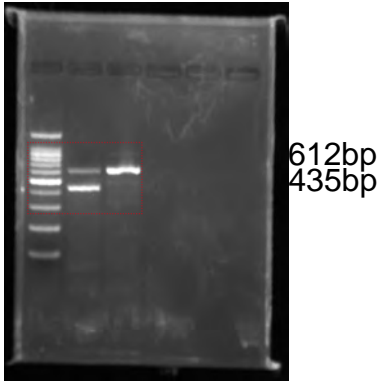

Ngf<sup>ms/+</sup>  
+/+

Supplement: Supplementary file 12 — Unprocessed images. [file 41556_2023_1284_MOESM12_ESM.pdf]

Unprocessed images in Extended Data Figure 3

**b**

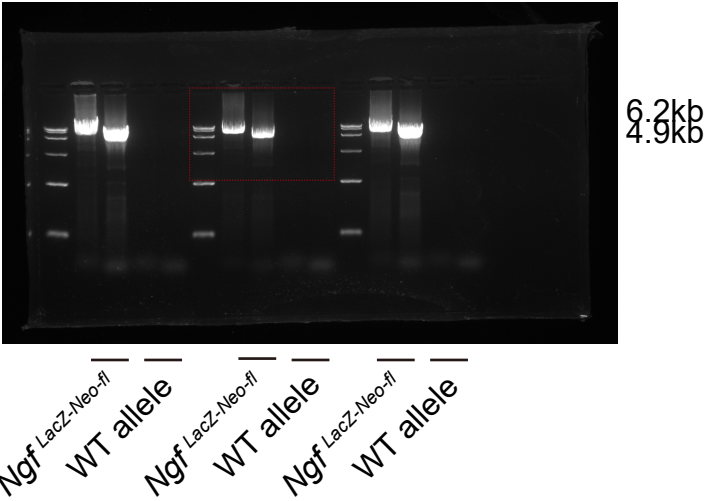

**c**

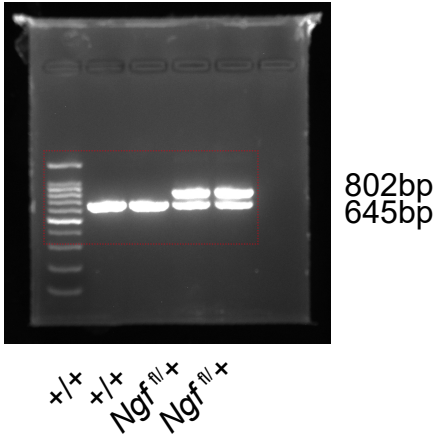

Supplement: Supplementary file 13 — Unprocessed images. [file 41556_2023_1284_MOESM13_ESM.pdf]

Unprocessed images in Extended Data Figure 8

j

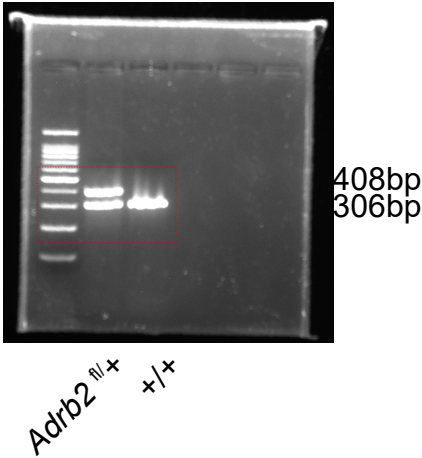

l

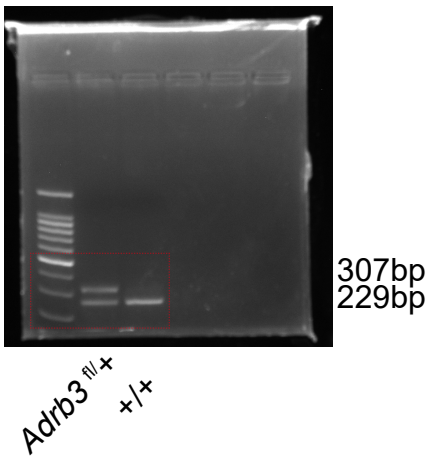

Supplement: Supplementary file 19 — Unprocessed images. [file 41556_2023_1284_MOESM19_ESM.pdf]
